# Pleiotropic effect of chromosome 5A and the *mvp* mutation on the metabolite profile during cold acclimation and the vegetative/generative transition in wheat

**DOI:** 10.1186/s12870-014-0363-7

**Published:** 2015-02-19

**Authors:** Zsófia Juhász, Ákos Boldizsár, Tibor Nagy, Gábor Kocsy, Ferenc Marincs, Gábor Galiba, Zsófia Bánfalvi

**Affiliations:** NARIC Agricultural Biotechnology Institute, Szent-Györgyi A. u. 4, 2100 Gödöllő, Hungary; Department of Plant Molecular Biology, Agricultural Institute, Centre for Agricultural Research, Hungarian Academy of Sciences, Brunszvik u. 2, 2462 Martonvásár, Hungary; Doctoral School of Animal and Agricultural Environmental Sciences, Department of Plant Sciences and Biotechnology, University of Pannonia Georgikon Faculty, Deák Ferenc u. 16, 8360 Keszthely, Hungary

**Keywords:** GC-MS, Metabolite profiling, *Triticum aestivum*, *Triticum monococcum*, Vernalisation

## Abstract

**Background:**

Wheat is the leading source of vegetable protein in the human diet, and metabolites are crucial for both plant development and human nutrition. The recent advances in metabolomics provided an opportunity to perform an untargeted metabolite analysis in this important crop.

**Results:**

Wheat was characterised at the metabolite level during cold acclimation and transition from the vegetative to the generative phase. The relationship between these changes and chromosome 5A and the maintained vegetative phase (*mvp*) mutation was also investigated. Samples were taken from the shoots and crowns during four developmental stages: plants grown at 20/17°C, after cold treatment but still during the vegetative phase, at the double ridge and during spikelet formation. The levels of 47 compounds were identified by gas chromatography-mass spectrometry, of which 38 were annotated. The cold treatment, in general, increased the concentrations of osmolites but not in all lines and not equally in the shoots and crowns. The accumulation of proline was not associated with the vernalisation process or with frost tolerance. The *mvp* mutation and chromosome 5A substitutions altered the amounts of several metabolites compared to those of the Tm and CS, respectively, during each developmental stage. The Ch5A substitution resulted in more substantial changes at the metabolite level than did the Tsp5A substitution. While Ch5A mainly influenced the sugar concentrations, Tsp5A altered the level of tricarboxylic acid cycle intermediates during the vegetative/generative transition. A much higher trehalose, proline, glutamine, asparagine, and unidentified *m*/*z* 186 content was detected in crowns than in shoots that may contribute to the frost tolerance of crowns.

**Conclusions:**

Substantial influences of chromosome 5A and the *mvp* mutation on metabolism during four different developmental stages were demonstrated. The distinct and overlapping accumulation patterns of metabolites suggest the complex genetic regulation of metabolism in the shoots and crowns.

**Electronic supplementary material:**

The online version of this article (doi:10.1186/s12870-014-0363-7) contains supplementary material, which is available to authorized users.

## Background

Cereals are significant sources of food and animal feed, constituting more than 50% of the worldwide crop production. Globally, wheat is the leading source of vegetable protein in the human diet (http://faostat.fao.org/).

*Triticum aestivum* (common wheat or bread wheat) is a cultivated, allohexaploid wheat species with six sets of chromosomes (AA, BB, and DD), two sets from each of three different species [1]. Common wheat is an annual plant with several varieties of two classes: winter and spring wheat. Winter wheat is sown in the Northern Hemisphere from September to December, sprouts before freezing occurs and, as a result of shortening day lengths and decreasing temperatures, cold acclimates. This process is double faced: while it results in increased freezing tolerance (cold hardiness), it fulfils the vernalisation requirement [2]. Spring cereals are usually planted in spring and do not have a vernalisation requirement [3,4].

Natural variation in the vernalisation requirement in the temperate cereals is mainly associated with allelic differences in the *VRN1*, *VRN2* and *VRN3* vernalisation genes. *VRN1* encodes a MADS-box transcription factor with high similarity to the *Arabidopsis* meristem identity genes *APETALA1*, *CAULIFLOWER* and *FRUITFULL*, which regulate the transition of the vegetative shoot apical meristem to the reproductive phase [5]. Winter genotypes that are maintained under continuous cold after an initial increase in freezing tolerance exhibit a progressive decrease in their cold acclimation ability after the shoot apical meristem advances to the double ridge stage [6-8]. The expression of the *VRN1* gene gradually increased in winter genotypes during cold acclimation, while the cold-regulated genes (*COR*), which were positively associated with freezing tolerance, were down regulated [9]. Dhillon et al. [10] demonstrated a direct connection between the two processes.

*T. monococcum* (einkorn wheat) is one of the earliest cultivated forms of wheat and is a diploid species [11]. In this species, a radiation-induced deletion encompassing the *VRN1* gene resulted in a mutant that was designated maintained vegetative phase (*mvp*), as homozygous *mvp*/*mvp* plants never flower, whereas plants carrying at least one functional *VRN1* copy (*Mvp*/-) exhibit normal flowering. The *Mvp*/- plants show reduced freezing tolerance and reduced transcript levels of several cold-induced genes relative to those of the deletion mutant (*mvp*/*mvp*) plants [10,12].

Because *T. aestivum* is hexaploid, its vital genes are replicated, allowing Sears [13] to develop a series of nullisomic lines (i.e., lines lacking one of the normal chromosomal pairs) from Chinese Spring (CS), a freezing-susceptible spring wheat. This genetic stock made possible the creation of single chromosome substitution lines. The genetic composition of the homologous alleles of *VRN1* genes in CS is *vrn*-*A1*, *vrn*-*B1* and *Vrn*-*D1*. Because CS carries the single dominant vernalisation-insensitive *Vrn*-*D1* allele, CS is spring wheat [14,15]. In a study using a series of 5A substitution lines in CS, the most vernalisation insensitive *Vrn1* allele was found on chromosome 5A of a *T. spelta* accession [16]. By replacing chromosome 5A of CS with the corresponding chromosome from a spring-type *T. spelta* accession [CS(Tsp5A)] conferred upon CS not only earlier ear-emergence [16] but also additional freezing sensitivity [14]. Cheyenne (Ch) is a winter wheat carrying recessive *VRN1* alleles (*vrn*-*A1*, *vrn*-*B1*, and *vrn*-*D1*). Replacing chromosome 5A of CS with the corresponding chromosome from Ch, [CS(Ch5A)] greatly increased the freezing tolerance of CS [17,18]. The freezing tolerance locus (*FR2*) on wheat 5A chromosomes contains a cluster of *CBF* (C-repeat binding factor) genes [19]. The increased freezing tolerance of the CS(Ch5A) substitution line is due to the increased transcription level of winter wheat Ch *CBF* genes in the CS genetic background [20].

The presence of Ch chromosome 5A in the CS background significantly elevated the sugar concentration, particularly that of sucrose and fructan, thereby resulting in increased osmotic potential and improved the freezing tolerance of the plants [21]. The accumulation of osmotic stress-induced free amino acids and polyamines is also regulated by chromosome 5A [22,23]. The role of chromosome 5A in the control of gene expression during cold hardening was demonstrated via a comparison of the cold-induced changes in the transcriptomes of CS and the two substitution lines. The genes that are affected by chromosome 5A encoded proteins that are involved in transcriptional regulation, defence processes and carbohydrate metabolism [24].

Currently, metabolomics approaches have enabled the parallel assessment of the levels of a broad range of metabolites and have had great value in both phenotyping and diagnostic analyses in plants [25]. Based on our previous finding at the transcript profile level [24], we supposed that chromosome 5A affects plants also at metabolite profile level. In this study, we used the tools of metabolomics to identify differences that are characteristic for cold acclimation and transition from the vegetative to the reproductive phases both in a *T. monococcum* accession KU 104-1 and *T. aestivum* ssp. *ae*. cv. Chinese Spring and investigated the involvement of chromosome 5A and its *VRN1* region in these two processes.

## Results

### Experimental design

A comparative metabolite analysis of the cultivated, moderately freezing-sensitive *T. aestivum* cv. Chinese Spring (CS) and its two chromosome 5A substitution lines, the freezing-tolerant CS(Ch5A) and the freezing-sensitive CS(Tsp5A), together with *T. monococcum* (Tm) and its *mvp* mutant was designed. The putative homozygous *mvp* mutant lines were selected by genotyping because they could not be propagated due to the lack of flowering, and their phenotype was not different from that of wild type and heterozygous plants at the seedling stage. The homozygous *mvp* mutants were assorted by PCR, in which the primers were designed to amplify the missing *VRN1* gene; consequently, no amplicons could be detected.

**Figure 1:**
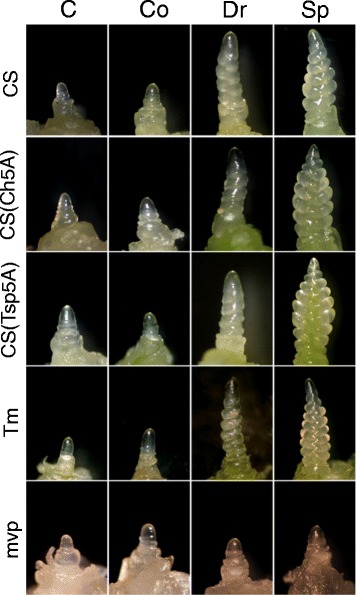
**Apices of the plants during four different developmental stages.** CS, Chinese Spring; CS(Ch5A) Chinese Spring with Cheyenne chromosome 5A substitution; CS(Tsp5A), Chinese Spring with *Triticum spelta* chromosome 5A substitution; Tm, *Triticum monococcum*; mvp, *mvp* mutant of *Triticum monococcum*; C, control; Co, after cold treatment but still in vegetative phase; Dr: double ridge formation stage; Sp, spikelet formation stage.

**Figure 2:**
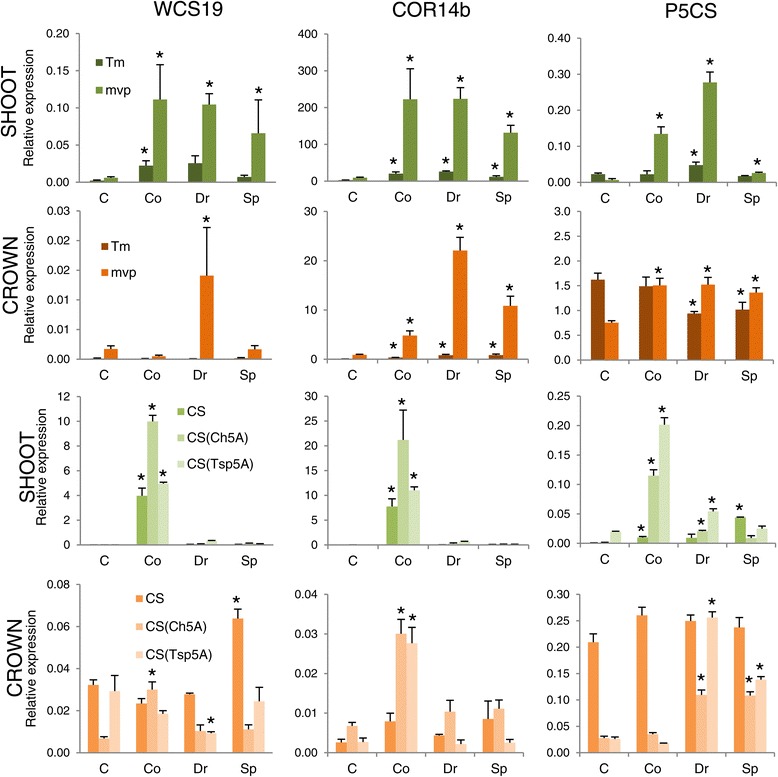
**qRT**-**PCR analysis of** ***WCS19***, ***COR14b*** **and** ***P5CS*** **genes across developmental stages.** The values represent means ± SD of nine plants in three parallels. Asterisks indicate values determined by *t* test to be significantly different from the C stage in each individual line (*P* < 0.01). CS, Chinese Spring; CS(Ch5A) Chinese Spring with Cheyenne chromosome 5A substitution; CS(Tsp5A), Chinese Spring with *Triticum spelta* chromosome 5A substitution; Tm, *Triticum monococcum*; mvp, *mvp*-*2* mutant of *Triticum monococcum*; C, control; Co, after cold treatment but still in vegetative phase; Dr: double ridge formation stage; Sp, spikelet formation stage; Sh, shoot; Cr, crown.

Metabolite profiling was performed on soil-grown plants in four different stages: (1) samples were collected before cold treatment from nine plants in three parallels from 21-day-old plants that were grown at 20/17°C (control, C) with the exception of CS(Tsp5A) because this genotype developed faster than did the others [16]; therefore samples from this line were collected at day 14^th^; (2) after 10 (Tm and *mvp*) and 14 days (CS lines) of cold treatment at 3°C in vegetative phase (Co); (3) in double ridge stages (Dr); and (4) when the spikelets (Sp) formed (Figure 1). The entire experiment was repeated three times. The plants with the CS, CS(Ch5A) and CS(Tsp5A) genotypes reached the Dr stage after 71 ± 7, 80 ± 7 and 49 ± 9 days and the Sp phase after 90 ± 7, 95 ± 7 and 68 ± 7 days from germination, respectively. From the Tm and *mvp* plants, Dr and Sp samples were collected after 42 ± 7 and 73 ± 7 days, even though in the case of *mvp* plants, no phenotypic alteration of the apices occurred (Figure 1). Because it was clear that cold-responsive genes are differentially expressed between different tissues [26], both the shoots and crowns were investigated. Given the documented response of *WCS19*, *COR14b* and *P5CS* genes to abiotic stresses [10,27,28] we have chosen these genes as molecular indicators of the cold treatment. Figure 2 shows that, in general, the cold up-regulated the expression of all three tested genes, however, with different extents in different lines, organs and developmental phases. The level of *WCS19* and *COR14b* expression, for example, was much higher in shoots than in crowns and was limited to the vegetative stage in shoots of CS lines. The *mvp* mutation caused the up-regulation of all three genes in shoots during the cold treatment. The level of *WCS19* and *COR14b* expression was also higher in *mvp* than in Tm crowns during the entire growth phase. This is in line with a recent observation that the *mvp* mutation reveals important changes in global gene expression [29].

### Identification of metabolites by gas chromatography-mass spectrometry (GC-MS)

**Figure 3:**
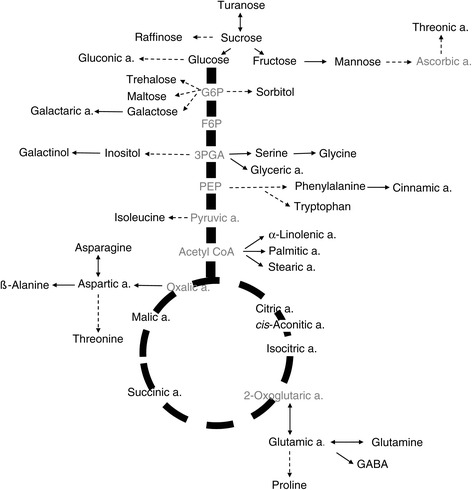
**Simplified scheme of primary metabolite synthesis.** The black letters depict the compounds that were detected, while grey letters depict the compounds that were not detected in the pathway.

The polar primary metabolite composition of the collected shoot and crown samples was analysed by GC-MS. The data were obtained from three replicate experiments except for the *mvp* crowns, from which only two biological repeats were analysed in the C and Co stages. In total, 354 datasets were generated. In summary, the levels of 47 compounds were measured. The main classes of detected compounds included amino acids, organic acids, sugars and sugar alcohols. In addition to the 38 identified components, nine unidentified peaks were detected in various samples (Table 1 and Additional files 1, 2, 3, 4, 5 and 6). A simplified scheme of the biosynthesis of the identified primary metabolites is presented in Figure 3.

**Table 1:** **Compounds detected in wheat samples by GC**-**MS analysis**

**Compound class**	**Metabolites**
**Amino acid** ^**a**^	ß-alanine, γ-aminobutyric acid, asparagine, aspartic acid, glutamic acid, glutamine, glycine, isoleucine, 5-oxoproline^b^, phenylalanine, proline, serine, threonine, tryptophan
**Sugar** ^**a**^	fructose, galactose, glucose, maltose, mannose, ribose, raffinose, sucrose, trehalose, turanose
**Organic acid** ^**b**^	*cis*-aconitic acid, cinnamic acid, citric/ isocitric acid, galactaric acid, glyceric acid, malic acid, succinic acid, threonic acid
**Sugar alcohol** ^**b**^	galactinol, inositol, sorbitol
**Fatty acid** ^**b**^	palmitic acid, stearic acid, α-linolenic acid
**Unidentified**	*m*/*z* 116, *m*/*z* 245, *m*/*z* 160, *m*/*z* 234, *m*/*z* 186, *m*/*z* 204,205, *m*/*z* 204,191, *m*/*z* 387,299,357, *m*/*z* 597

^a^With the exception of 5-oxoproline, identification of amino acids and sugars was based on authentic standards.

^b^Organic acids, sugar alcohols, fatty acids and 5-oxoproline were identified by searching the NIST 11 mass spectral database.

**Figure 4:**
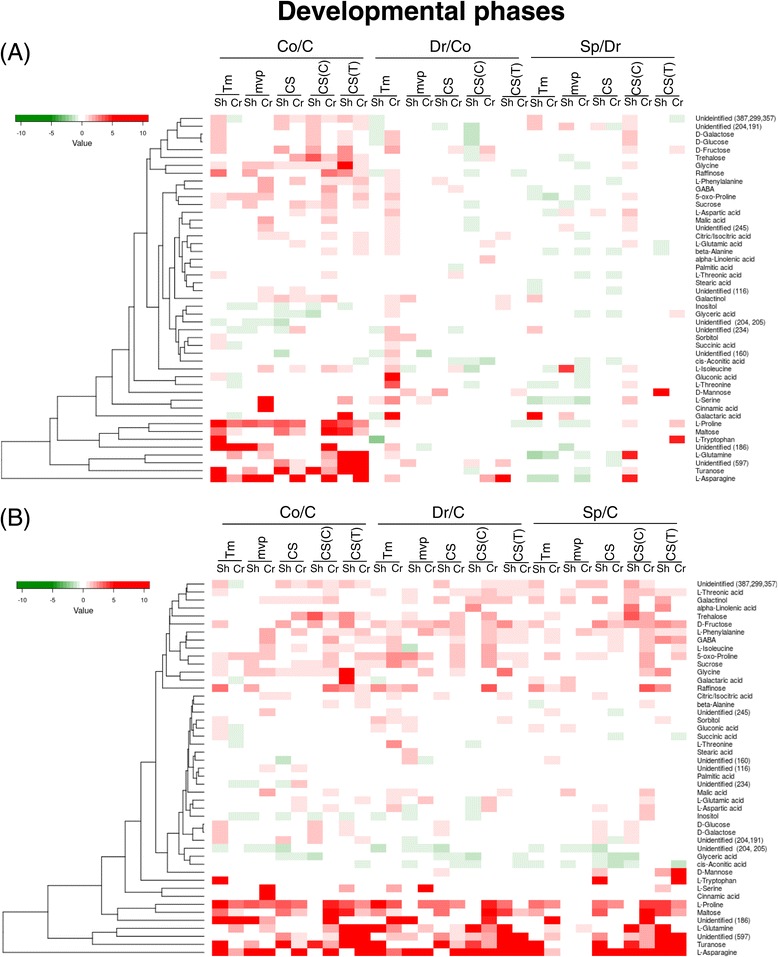
**Clustered heat**-**map of the metabolite changes related to developmental phases.** The data were derived from three independent experiments. In each experiment, the samples were collected from a group of nine plants in three replicates except for the *mvp* crowns during the C and Co stages, in which the samples could be collected only in two experiments. Only those compounds that showed significant changes (one-way MANOVA, *P* < 0.05) between the control and cold-treated samples (Co/C), cold-treated samples in the vegetative and double ridge formation stages (Dr/Co) and double ridge formation via spikelet formation stages (Sp/Dr) in all of the experiments are included in the heat-map. The red colour depicts increases, while the green colour depicts decreases from one stage to another **(A)** and from a given stage to the control stage **(B)** in concentrations of the compounds that are listed in the figure. The scale is in log2. 5-Oxo-proline was derived from glutamic acid during the methoximation step. CS, Chinese Spring; CS(C) Chinese Spring with Cheyenne chromosome 5A substitution; CS(T), Chinese Spring with *Triticum spelta* chromosome 5A substitution; Tm, *Triticum monococcum*; mvp, *mvp*-*2* mutant of *Triticum monococcum*; C, control; Co, after cold treatment but still in vegetative phase; Dr: double ridge formation stage; Sp, spikelet formation stage.

**Figure 5:**
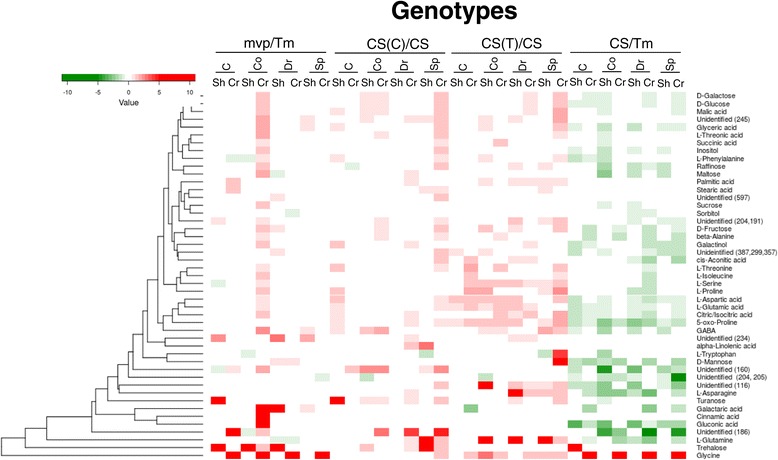
**Clustered heat**-**map of the metabolite changes related to genotypes.** The data are the same as those presented in Figure 4. The scale is in log2.

**Figure 6:**
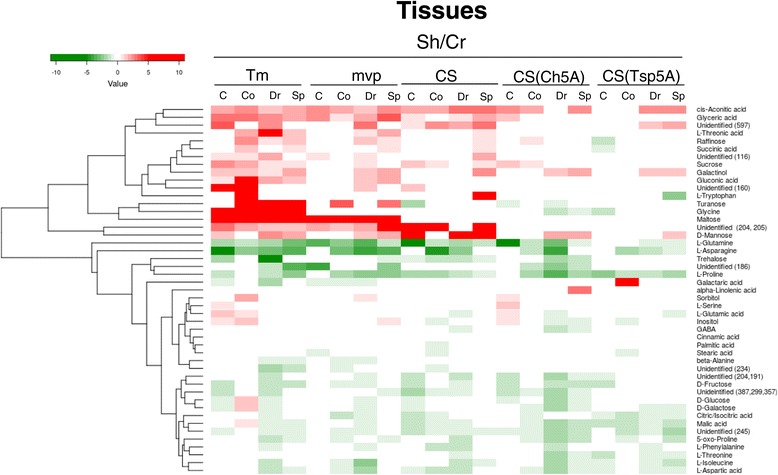
**Clustered heat**-**map of the metabolite differences between shoots and crowns.** The data are the same as those presented in Figure 4. The scale is in log2.

Significant differences in the primary metabolite composition between the different developmental phases were determined by a one-way MANOVA. The means of three parallels in each experiment were calculated from the control and samples representing the different developmental phases, and the ratios of the means were determined. Compounds showing the same tendency of changes between the control and samples of different developmental phases in all three of the consecutive experiments were included into the heat-maps (Figures 4, 5 and 6). In case of *mvp* crowns, from which samples could be collected only in two experiments, the means of the two data sets were used as a third data set.

### Metabolite changes that were related to developmental phases

In general, both in the shoots and crowns, the cold increased the amounts of primary metabolites (Figure 4A and B). Except for the CS(Ch5A) shoots, a significant increase in the proline concentration was observed in all of the samples. Characteristic increases were also detected in the fructose, trehalose, raffinose, maltose, turanose, sucrose, glucose, galactinol, GABA, glutamine and asparagine contents as well as in the concentration of two unidentified compounds with *m*/*z* 186 and 597, albeit not in all lines and tissues. In contrast, except for Tm, a decrease in the inositol content was detected in all the cold-treated shoot samples.

No compound with the same tendency of changes by double ridge (Dr) formation compared to the Co stage in the five wheat genotypes was identified. Broadly, however, except for the Tm crowns, the majority of these changes were reductions (Figure 4A). Compared to the C stage, however, several compounds, and especially those induced by cold, were present in higher amounts in each genotype and tissue (Figure 4B).

The spikelet formation, with a few exceptions, was characterised by decreases in the metabolite concentrations, especially in the crowns, where almost all of the detected changes were negative compared to the previous developmental stage (Figure 4A). Nevertheless, compared to the C stage, concentration of the cold-induced metabolites was still relatively high (Figure 4B).

### Metabolite changes that were related to the *mvp* mutation and chromosome 5A

Substantial differences (log2 > 1 or log2 < -1) between Tm and *mvp* were detected in most of the stages in both the shoots and crowns (Figure 5). For example, much more glycine was detected in *mvp* than in Tm crowns in all stages and much more trehalose was detected in *mvp* than in Tm shoots in C, Co and Dr stages. The *mvp* mutation also increased the turanose content in C, the gluconic acid content in Co, and the galactaric acid concentration in Co and Dr stages. The amount of the unidentified compound *m*/*z* 234 was also much higher in three out of eight different *mvp* than in Tm samples. Nevertheless, based on a pairwise correlation analysis majority of the metabolites coordinately changed during development in Tm and *mvp* (Additional file 7).

The substitution of chromosome 5A in CS by either Ch5A or Tsp5A resulted in substantial differences in the metabolite levels during each stage (Figure 4A). For example, the concentrations of 12 metabolites decreased in CS(Ch5A) but not in the CS shoots during the double ridge formation. Seven of these metabolites were sugars, i.e., galactose, glucose, fructose, trehalose, raffinose, sucrose and turanose. During spikelet formation, changes in the metabolite content were very different in the CS, CS(Ch5A) and CS(Tsp5A) in both the shoots and crowns. The pairwise correlation analysis showed that the substitution of chromosome 5A of CS by Ch5A resulted in more changes at the metabolite level than did that by Tsp5A (Additional file 7), and while Ch5A influenced mainly the sugar concentrations, Tsp5A altered the level of TCA-cycle intermediates (Additional file 8).

Chromosome 5A substitution resulted in elevation of metabolite concentration both in shoots and crowns of the CS line (Figure 5). Ch5A highly increased the amount of unidentified *m*/*z* 186, glutamine, trehalose and α-linolenic acid in certain tissues and stages. Genotypes CS, CS(Ch5A) and CS(Tsp5A) differ in frost sensitivity. Correlation of metabolite concentrations with the frost tolerance of the lines was investigated. Out of the cold-induced compounds, higher amount of glucose, trehalose, turanose, galactinol, and unidentified *m*/*z* 186 were detected in the frost tolerant CS(Ch5A), but not in the most freezing sensitive CS(Ch5A), than in the shoots or crowns of the CS line with moderate freezing sensitivity. Thus we concluded that these compounds may contribute to the frost tolerance of the CS(Ch5A) line.

As expected, the largest differences were detected between the two *Triticum* species, *T. monococcum* and *T. aestivum* (Additional file 7). Concentration of most of the metabolites was lower in CS than in Tm, except of trehalose and glycine, which were present in much higher amounts in Tm shoots in C stage and in crowns during the whole life cycle, respectively (Figure 5).

### Differences in metabolite composition of shoots and crowns

Ganeshan et al. [26] reported that *COR* genes are differentially expressed in leaf and crown tissues of wheat during an extended low temperature acclimation regimen. Thus we supposed that there are characteristic differences also in the metabolite composition of shoots and crowns. Figure 6 shows that there are really substantial differences in concentration of certain compounds and these differences are bigger in *T. monococcum* than in *T. aestivum*. The concentration of maltose is much higher in Tm shoots than in crowns in each developmental stage and this is not influenced by the *mvp* mutation. In general, the level of *cis*-aconitic acid and glyceric acid is higher, while the level of glutamine, asparagine, proline, trehalose and unidentified *m*/*z* 186 is lower in shoots than in crowns.

## Discussion

Under cold stress conditions, plants accumulate compatible solutes, such as GABA, galactinol, maltose, proline, raffinose, sucrose and trehalose [30]. The results of this study demonstrate that the levels of all these metabolites increased in response to the cold treatment but not in all of the lines and not equally in the shoots and crowns (Figure 4). The most general increase was detected in the proline concentration as, except for the CS(Ch5A) shoots, this compound accumulated in both the shoots and crowns of each line in all three of the experiments. *Mvp* is a vernalisation mutant of Tm, while CS, CS(Ch5A) and CS(Tsp5A) differ in frost sensitivity. We found no association between the accumulation of proline and the vernalisation or freezing tolerance. Although a large body of data suggests a positive correlation between proline accumulation and plant stress tolerance [31], negative examples also exist. No correlation could be established, for example, between variations in the proline pool during cold exposure and the level of freezing tolerance for *Arabidopsis* and *Thellungiella* sp. [32]. The annual temperate wild grass *Brachypodium* is a model to study the response of temperate cereals to their environment. Although exposure to cold temperatures triggers the accumulation of proline in *Brachypodium*, the size of the proline pool of a given accession cannot be used to accurately predict its freezing tolerance behaviour [33]. Comparing three wheat cultivars with different freezing tolerances, the proline content increased in all of the cultivars after one week of cold acclimation, but a prolonged cold acclimation resulted in different profiles: no further increase occurred in the most sensitive cultivar, while an additional increase occurred in the other two cultivars [34]. We analysed the metabolite composition of the shoots and crowns after different durations of cold treatment; however, we detected a slight increase in the proline content compared to the previous stage only in the Tm crowns at double ridge formation and in the CS(Ch5A) and CS(Tsp5A) shoots when the spikelets were formed (Figure 4A). Thus, even the time course of proline content did not show a correlation with the freezing tolerance of the wheat genotypes in our experiment.

In plants, proline is synthesized mainly from glutamate, which is reduced to glutamate-semialdehyde by the pyrroline-5-carboxylate synthetase (P5CS) [31]. We have tested the level of *P5CS* expression and found that the cold up-regulated it in each line, both in shoots and crowns, however, with different extents and in different stages (Figure 2). The level of *P5CS* expression did not correlate with the proline concentration (Figures 4, 5 and 6) suggesting that, like in other plant species [31], the proline concentration is regulated by the interplay of biosynthesis, degradation and transport processes also in wheat.

The existence of positive correlations between the level of freezing tolerance of cereals and their capacities to accumulate water-soluble carbohydrates including sucrose and glucose was also previously observed [21,35-37]. The observed changes in the metabolite levels in these cases may derive from the activation of the related genes which was observed in a previous study investigating the transcriptome of the cold-treated CS and the substitution lines [24]. The transcript level of the genes encoding sucrose synthase 1 (gb|AL506883), sucrose synthase 2 (gb|BQ663764), and β-glucosidase (gb|BQ659818) was increased by cold treatment in the shoots and a corresponding alteration was shown in sucrose and glucose levels in the present study (Figure 4). In addition, similarly to the amount of the glucose (Figure 5), the transcription of the β-glucosidase gene was also greater in CS(Ch5A) than in CS and CS(Tsp5A) [24].

Turanose is an analogue of sucrose that was previously described as a sugar and that is not synthesised or metabolised by higher plants; however, turanose elicits responses that are distinctly different from those of metabolisable sugars and that are perceived as stress-related stimuli [38]. Recently, however, turanose was detected in red-ripening strawberry fruits, tobacco leaves, the roots of herbal plants and *Agrostis* grass species, mandarin fruits, and the leaves and roots of barley [39-42]. Even the accumulation of turanose in barley upon salt stress was observed [43]. Based on a comparison with an authentic standard we detected turanose in the CS lines with an increasing concentration upon cold treatment (Figure 4). Nevertheless, the function of turanose in wheat calls for future research.

Our results show that, with the exception of the Tm crowns, in general, the concentrations of primary metabolites are lower both in the shoots and crowns upon the transition from the vegetative to reproductive phase than in the vegetative phase after cold treatment. The detected negative changes at the metabolite level (Figure 4A) coincide with the down-regulation of the cold regulated *COR* genes, which are positively associated with freezing tolerance [9]. One explanation for the negative changes in the metabolite concentrations might be the acclimatisation of plants to the cold environment. Another explanation might be the use of primary metabolites for flower formation. However, at the time of spikelet formation, negative changes in the concentrations of several metabolites were detected in the Tm as well as in the never-flowering *mvp* plants (Figure 4A), suggesting that the detected metabolites are used for vegetative growth rather than for spikelet formation. Nevertheless, majority of the cold-induced metabolites were present in higher concentrations during the transition from the vegetative to generative phase than in the control stage (Figure 4B).

The *mvp* mutation was generated by a nitrogen ion beam, and the deletion in this mutant encompasses the complete *VRN1* gene and other closely linked genes [44]. Metabolite profiling detected several differences in the metabolite composition of the Tm and *mvp* shoots and crowns at each developmental stage (Figure 5), which is in line with the influence of the *mvp* mutation on global gene expression [29], including the expression of *WCS19*, *COR14b* and *P5CS* genes (Figure 3). Because *VRN1* encodes a MADS-box transcription factor [5], these differences may be explained by the pleiotropic effect of the genes that are regulated by *VRN1*. It is also possible that the genes that are closely linked to *VRN1* are implicated in plant metabolism.

The influence of chromosome 5A, carrying the *VRN1* gene, on metabolism was further supported by the large number of differences that were detected between the metabolite contents of the CS, CS(Ch5A) and CS(Tsp5A) lines (Figure 5). Substitution of chromosome 5A of CS by Ch5A resulted in more changes at the metabolite level than did that by Tsp5A, and while Ch5A influenced mainly the sugar concentrations, Tsp5A altered the level of TCA-cycle intermediates (Additional files 7 and 8).

Large number of differences was detected between the metabolite composition of shoots and crowns (Figure 6). The crown is the most freeze-resistant part of the plant [45]. When the crown meristematic tissue is destroyed by freezing, the plants are unable to resume growth in the spring [46]. We found a much higher glutamine, asparagine, trehalose, proline and unidentified *m*/*z* 186 content in crowns than in shoots that may contribute to the frost tolerance of crowns.

## Conclusions

At the metabolite level, the differential accumulation of osmotically active solutes that were induced by cold treatment was observed in the shoots and crowns of five wheat genotypes. However, while the pool size of glucose, trehalose, turanose, galactinol and unidentified *m*/*z* 186 correlated with the freezing tolerance of *T. aestivum* Chinese Spring and its two chromosome 5A substitution lines, the proline content did not. The *mvp* mutation, encompassing the complete *VRN1* gene and several closely linked genes, resulted in several differences in the metabolite composition compared to that of the wild type *T. monococcum*, which might be explained by the pleiotropic effect of the genes that are regulated by the MADS-box transcription factor VRN1. The transition from the vegetative to generative phase reduced the level of several metabolites in the shoots and crowns. An interesting difference was observed between the two Chinese Spring substitution lines with different freezing tolerances: while the Cheyenne chromosome 5A influenced mainly the sugar concentrations, the *T. spelta* chromosome 5A altered mostly the level of TCA-cycle intermediates during the vegetative/generative transition. Experiments with more varieties would result in a better dissection and, most likely, a generalisation of this interesting finding related to chromosome 5A. A much higher trehalose, proline, glutamine, asparagine and unidentified *m*/*z* 186 content was detected in crowns than in shoots that may contribute to the frost tolerance of crowns. Since the concentration of the compound with *m*/*z* 186 is associated with frost tolerance identification of this compound would help to understand the mechanism of cold acclimation.

## Methods

### Plant materials

A specific genetic system consisting of the moderately freezing-sensitive *Triticum aestivum* ssp. *aestivum* cv. Chinese Spring (CS) wheat variety and two chromosome 5A substitution lines, the freezing-tolerant Chinese Spring (*Triticum ae*. ssp. *ae*. cv. Cheyenne 5A) [CS(Ch5A)] and the freezing-sensitive Chinese Spring (*Triticum ae*. ssp. *spelta* 5A) [CS(Tsp5A)], were used in the experiments [47-49]. In addition, *Triticum monococcum* KU 104-1 strain and its *mvp*-*2* mutant that remains indefinitely in the vegetative state were analysed in this study. The mutant was generated by ion beam radiation and has a large deletion that includes *VRN1* [12] and several flanking genes [44]. No phenotypical differences in shoots and crowns of the Tm and *mvp* mutant and those of the CS and its chromosome 5A substitution lines were detected [12,49].

### Plant growth conditions

After germination for 6 days (1 day at 22°C, 3 days at 4°C, and 2 days at 22°C), the seedlings were grown in wooden boxes (42 × 30 × 18 cm) in a 2:1:1 (volume) mixture of garden soil, humus and sand, respectively, for 21 days in a growth chamber (Controlled Env. Ltd., Winnipeg, Canada) at a 20/17°C day/night temperature and 75/70% relative humidity with 16 h illumination at 260 μmol m^−2^ s^−1^. The cold treatment was performed at 3°C until the appearance of the spike primordia without changing the other growth conditions. Sampling was performed at the end of the growth at 20/17°C, after 10 or 14 days at 3°C (the apex was still in the vegetative phase), at the double ridge stage (Dr) of the apex (start of vegetative/generative transition) and after the appearance of the spikelet primordia (Sp).

### Genotyping of the *mvp* mutant plants

Leaf samples of 100 mg were collected from the Tm and *mvp* mutant lines and smashed by TissueLyser (Qiagen) (shaking settings: 25 Hz, 1 min and 30 sec). The total DNA was isolated from crushed material by the ZenoGene^40^ DNA isolation kit (Zenon Bio Ltd.) and eluted in the last step in 200 μl of elution buffer. A total of 2 μl of DNA extract was used in the PCR experiments with the *VRN1*-specific primers VRN1-F: 5’-ACAAGAAAAACACTTGCAGAGAAGTTCAGC-3’ and VRN1-R: 5’-CATGGTAAATTACTCGTACAGCCATCTCAGC-3’. These primers amplify a 1084-bp fragment of the *VRN1* gene that is not present in the homozygous mutant. The PCR conditions were as follows: 40 cycle of 94°C for 20 sec, 61°C for 30 sec, and 72°C for 1 min and 20 sec. In the case of doubtful samples, the method of Dhillon et al. [10] was used with slight modifications in the final concentration of primers, i.e., 0.4 μM MVP_F18, 0.3 μM MVP_R22 and 0.2 μM MVP_R23 (MVP_F-18: 5′-AGCCACAAGAACCGGGACTA-3′, MVP_R-22: 5′-ATTCAAGCCCCAATGTTCTC-3′, and MVP_R-23: 5′-CCCAAACTTTGCGGTGTATC-3′).

### Quantitative RT-PCR (qRT-PCR)

Total RNA was isolated using the Direct-zol™ RNA Miniprep Kit (Zymo Research) as described by the manufacturer. Reverse transcription was carried out with M-MLV reverse transcriptase and Oligo(dT)_18_ primer (Thermo Scientific) using the method of the supplier. The transcript levels were determined with real-time RT-PCR using a CFX96 Touch™ Real-Time PCR Detection System (Bio-Rad). The phosphogluconate dehydrogenase Ta30797 served as a reference gene [50]. Primers used for detection of *WCS19*, *COR14b* and *P5CS* expression were described in [10,27,28].

### Extraction and GC-MS analysis of the polar metabolites from wheat

The shoot and crown samples were ground into a fine powder in liquid nitrogen. An extraction was performed according to Schauer et al. [51] using 100 mg of tissue powder. Ribitol was added to the samples as an internal standard. An aliquot of 150 *μ*l of extract was dried with 30 *μ*l of ribitol (20 mg ml^−1^). For methoxyamination, 40 *μ*l of methoxyamine hydrochloride (MEOX) that was dissolved at 20 mg ml^−1^ in pyridine was added to the dried extract and agitated for 90 min at 37°C. N-methyl-N-(trimethylsilyl) trifluoroacetamide (MSTFA) was used for the derivatisation (60 *μ*l, 30 min, and 37°C). The samples were analysed in the split mode in a quadrupole-type GC-MS system (Finnigan Trace/DSQ, Thermo Electron Corp., Austin, TX, USA) that was equipped with a 30-m capillary column (Rxi-5 ms, 0.25 mm ID, 0.25 μm df, Restek, Bellefonte, PA, USA). For GC-MS detection, an electron ionization system with ionization energy of 70 eV was used. Sample volumes of 1 μl were injected with a split ratio of 10 ml min^−1^ using the hot needle technique. The injection temperature was 230°C, and the temperatures of the interface and the ion source were set to 250°C. The carrier gas was helium, with a constant flow rate of 1 ml min^−1^. The temperature program was heating at 90°C for 2 min, followed by a 25°C min^−1^ oven temperature ramp to 165°C for 15 min. This ramp was followed by 6°C min^−1^ to 330°C. The system was temperature-equilibrated for 2 min at 90°C prior to injection of the next sample. The detection was performed in total ion chromatogram (TIC) positive mode. Mass spectra were recorded at 0.8170 scans sec^−1^ with an *m*/*z* 50-650 scanning range.

### Data analysis

The Thermo Scientific Xcalibur software was used for exporting the spectra and searching the NIST 11 mass spectral database. The NIST 11 is a fully evaluated collection of electron ionisation and mass spectra, with chemical and GC data, plus search software to identify the own unknown spectra. In addition, the sugars and amino acids were identified based on a comparison of the retention time and mass spectrum to an authentic standard that was analysed under identical conditions. A one-way MANOVA using the SPSS software package (SPSS Inc., an IBM Company) was used to determine whether there were significant differences between the metabolite compositions of independent groups of samples. For statistical analysis R (version 3.0.2) was used (http://www.r-project.org). Pairwise comparisons were performed using custom R scripts. Pearson correlation coefficients were calculated using R's built-in function cor. Student’s *t*-test was used to determine significant differences in gene expression at *P* < 0.01.

### Availability of supporting data

The datasets supporting the results of this study are included within the article and its additional files.

## Additional files

Additional file 1:
**Metabolite data file including a checklist and reporting list.** Reporting of the metabolite data follows the guideline of [52]. Additional file 2:
**Metabolites that were detected in the Tm.**
Additional file 3:
**Metabolites that were detected in the** ***mvp*** **.**
Additional file 4:
**Metabolites that were detected in the CS.**
Additional file 5:
**Metabolites that were detected in the CS(Ch5A).**
Additional file 6:
**Metabolites that were detected in the CS(Tsp5A).**
Additional file 7:
**Pairwise comparison of metabolite changes between** ***mvp*** **-Tm, CS(Ch5A)-CS, CS(Tsp5A)-CS and CS-Tm.**
Additional file 8:
**Simplified schemes of changes in primary metabolite content of CS(Ch5A) and CS(Tsp5A) lines from vegetative to generative phase.** In red are the compounds with increased concentrations upon the transition from the cold-treated vegetative stage to the double ridge formation stage (Co-Dr) and from the double ridge stage to the spikelet formation stage (Dr-Sp). In green are the compounds with decreased concentrations, while in black are the compounds with unchanged concentrations comparing the CS(Ch5A) to CS and the CS(Tsp5A) to CS. The grey letters depict the compounds that were not detected in the pathway. The concentration changes in shoots are circled, while those in the crowns are not circled. The concentration differences resulting from an increase in the CS but not in the CS(Ch5A) shoots are indicated by black letters and red circles. The concentration differences resulting from an increase in the shoots but a decrease in the crowns of CS(Tsp5A) are indicated by green letters and red circles. The differences resulting from a decrease in the CS crowns are depicted by green underlines. The schemes are based on the data that were presented in Figure 4.

## Abbreviations

C: Control
Ch5A: Cheyenne chromosome 5A
Co: Cold-treated during the vegetative phase
CS: Chinese spring
Dr: Double ridge formation
GC-MS: Gas chromatography-mass spectrometry
Sp: Spikelet formation
Tm: *Triticum monococcum*
Tsp5A: *Triticum spelta* chromosome 5A
VRN: MADS-box transcription factor regulating the vernalisation process
*mvp*: Maintained vegetative phase
