# Supplementary material for: Pleiotropic effect of chromosome 5A and the mvp mutation on the metabolite profile during cold acclimation and the vegetative/generative transition in wheat
Source: BMC Plant Biol. 2015 Feb 19;15:57. doi: 10.1186/s12870-014-0363-7 (PMC4349458; doi:10.1186/s12870-014-0363-7)
Supplement: Additional file 7: — Pairwise comparison of metabolite changes between mvp -Tm, CS(Ch5A)-CS, CS(Tsp5A)-CS and CS-Tm. [file 12870_2014_363_MOESM7_ESM.pptx]

## Slide 1
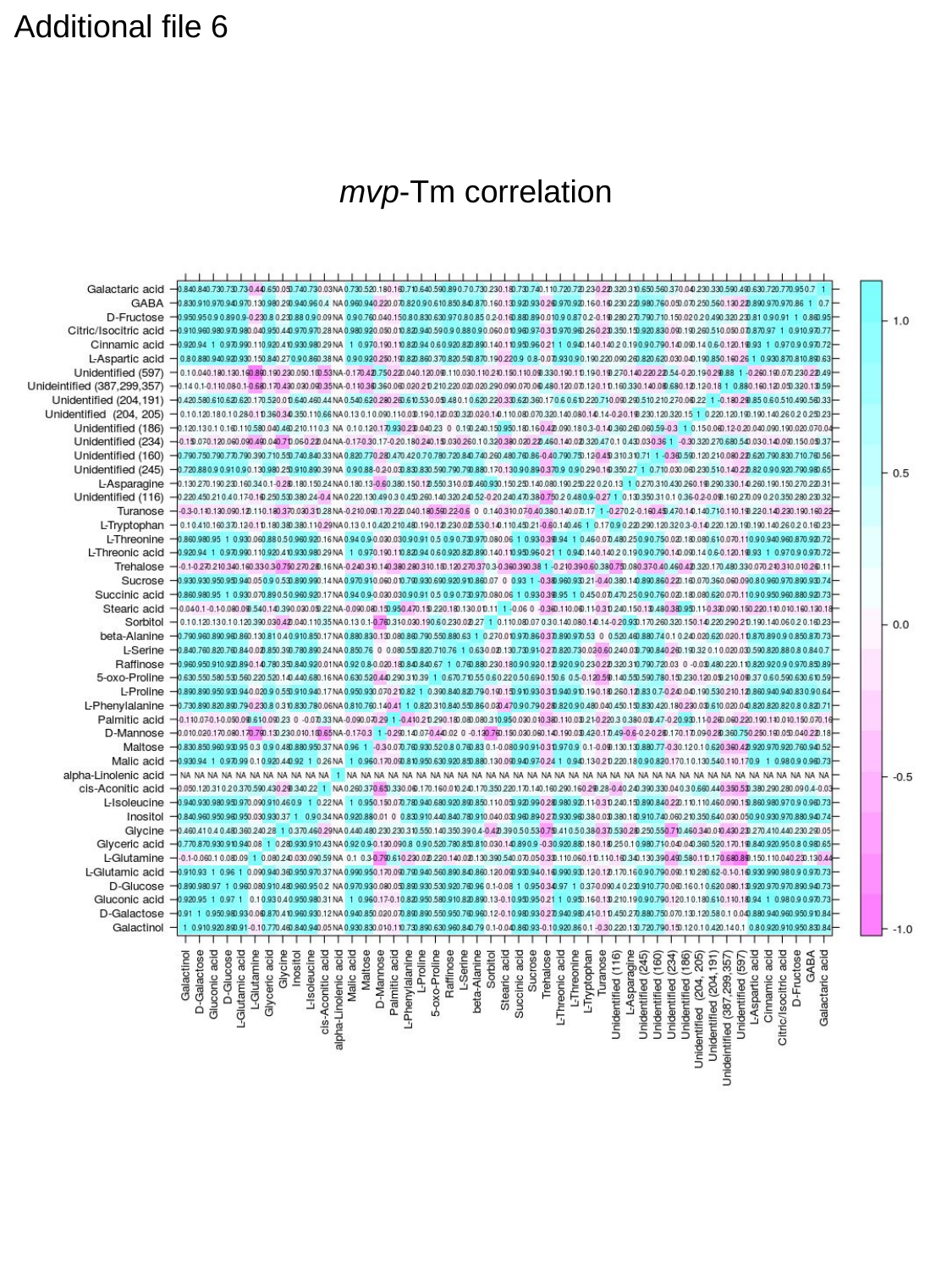

Additional file 6
mvp-Tm correlation

## Slide 2
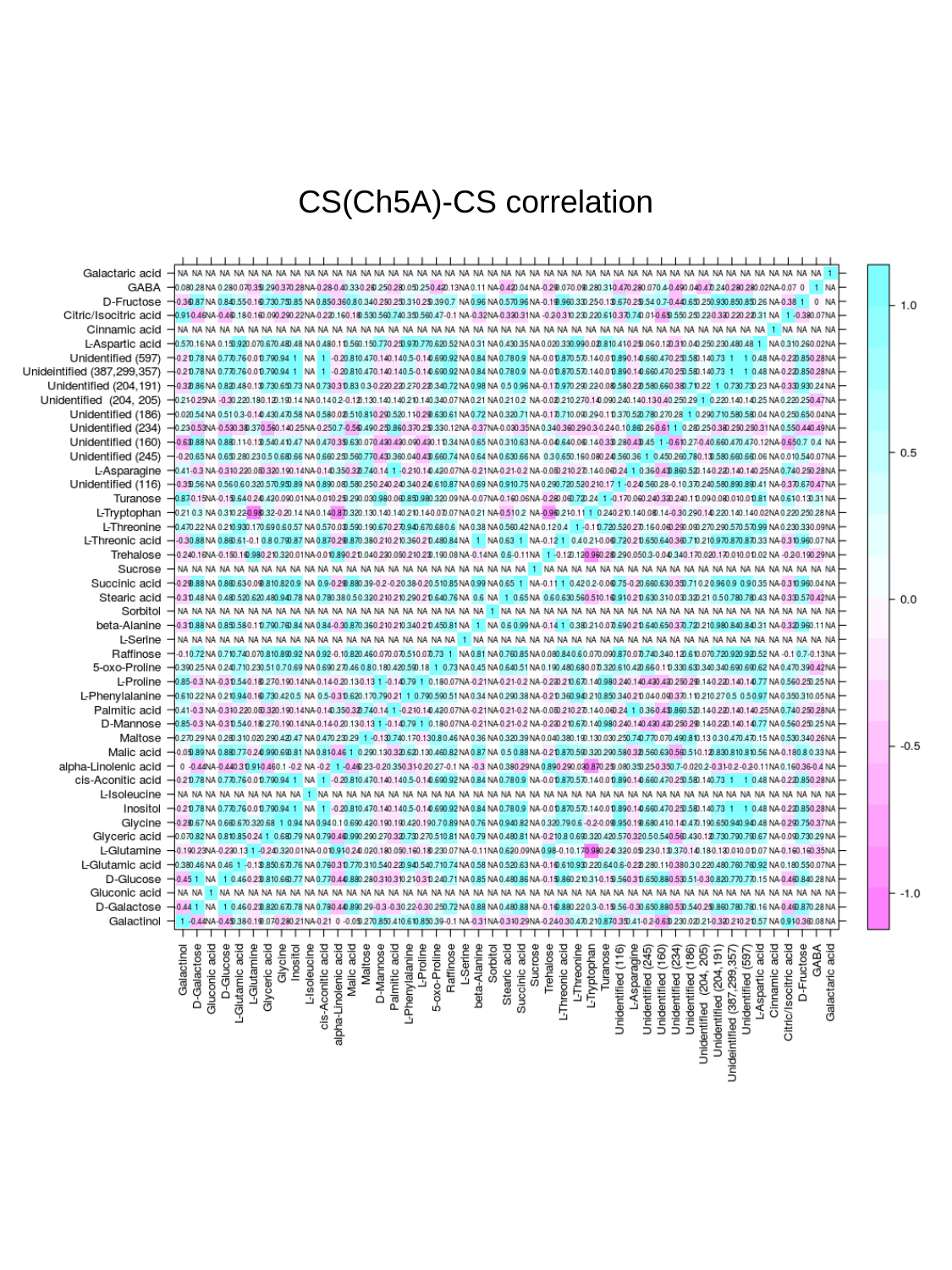

CS(Ch5A)-CS correlation

## Slide 3
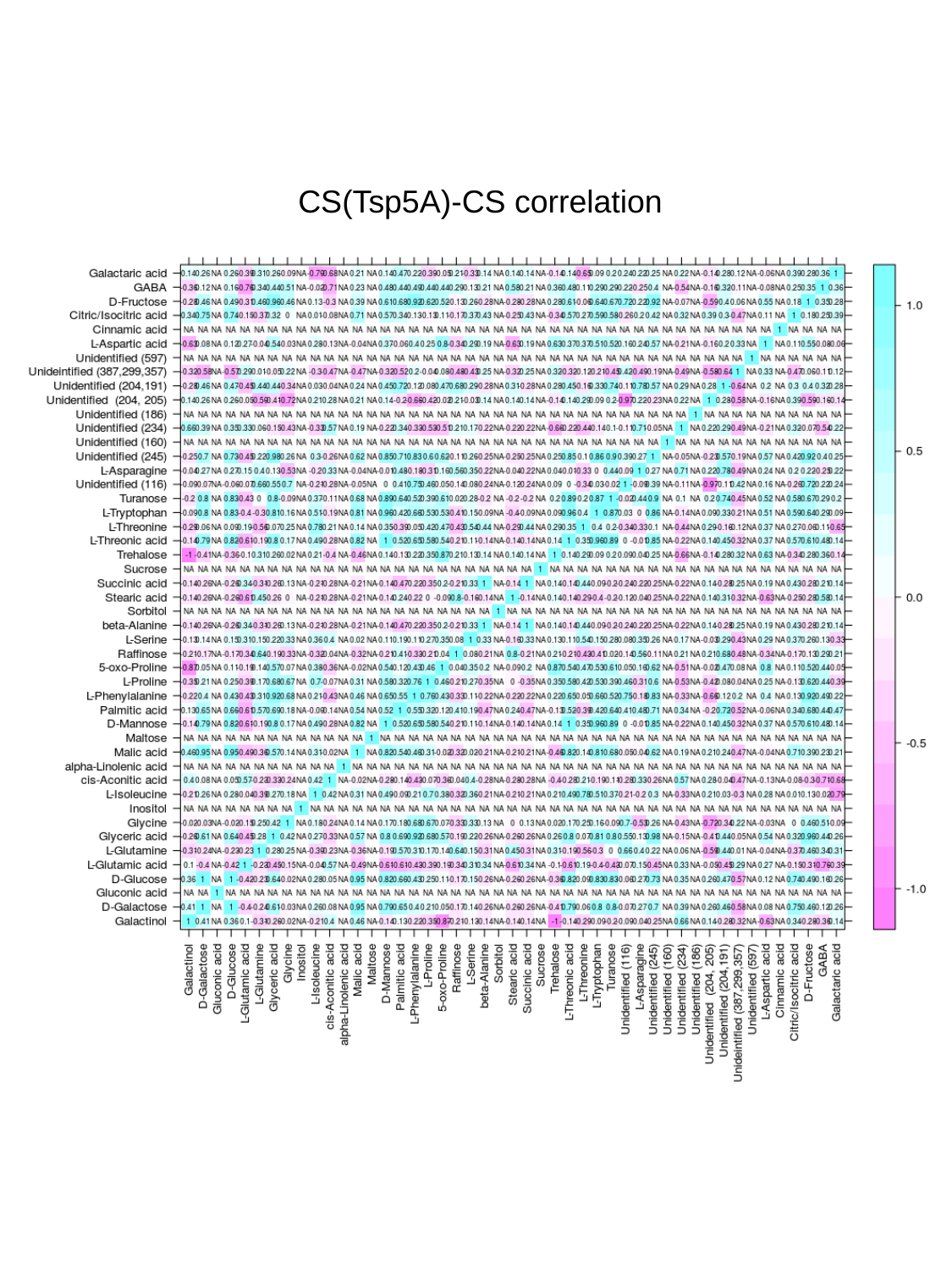

CS(Tsp5A)-CS correlation

## Slide 4
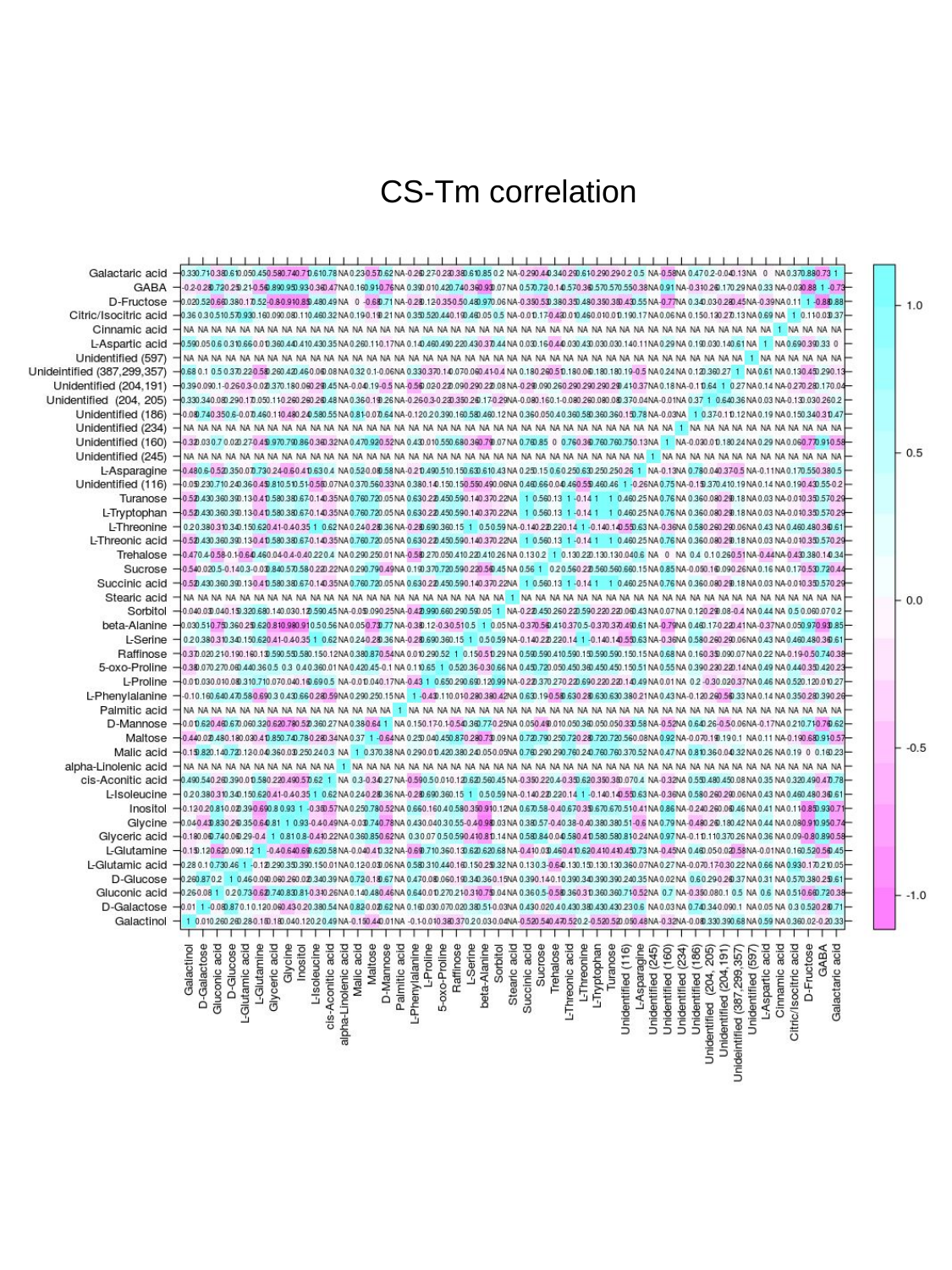

CS-Tm correlation
