# Supplementary figures and images for: Pleiotropic effect of chromosome 5A and the mvp mutation on the metabolite profile during cold acclimation and the vegetative/generative transition in wheat
Source: BMC Plant Biol. 2015 Feb 19;15:57. doi: 10.1186/s12870-014-0363-7 (PMC4349458; doi:10.1186/s12870-014-0363-7)

## Slide 1
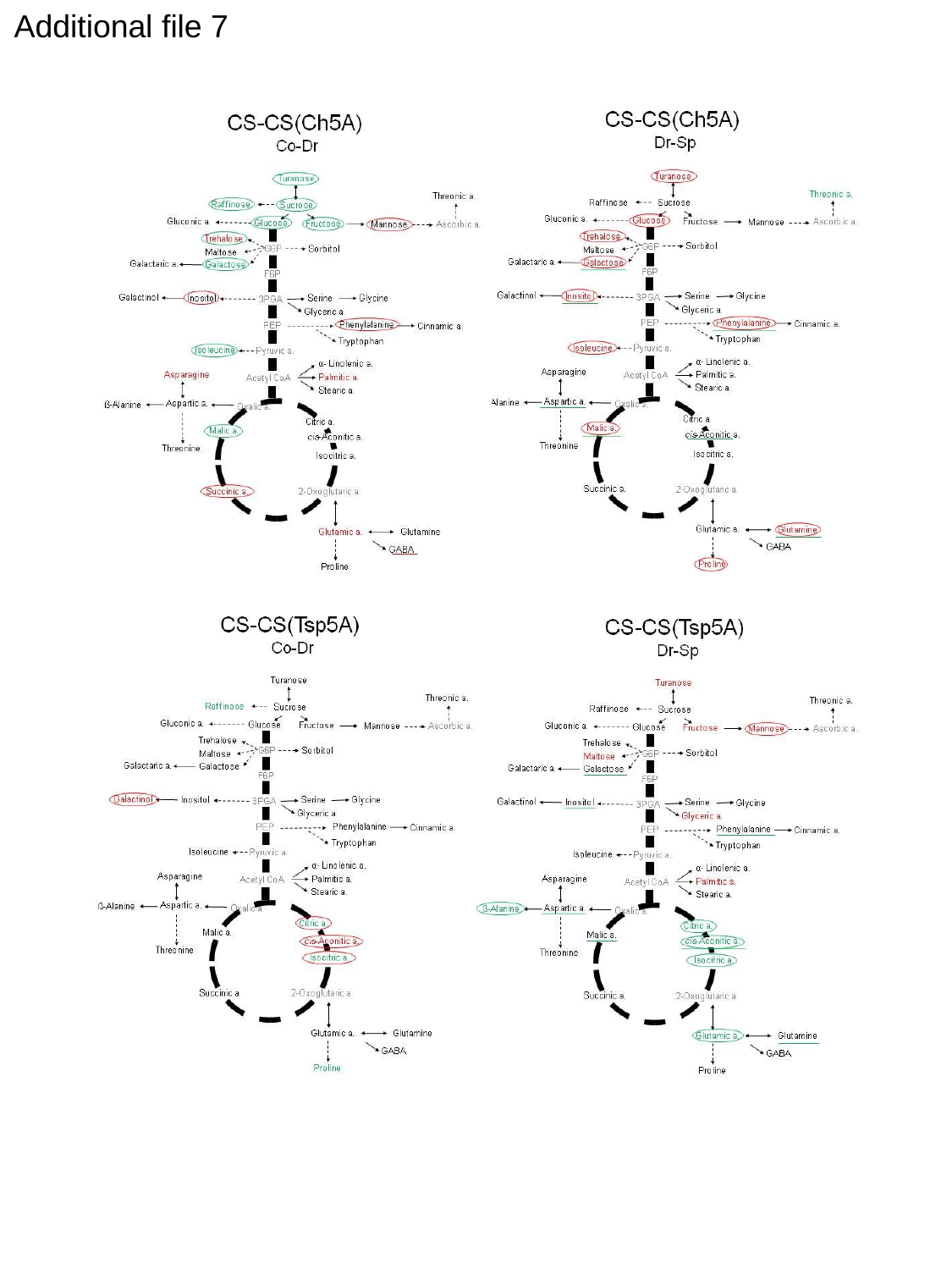

Additional file 7

Supplement: Additional file 8: — Simplified schemes of changes in primary metabolite content of CS(Ch5A) and CS(Tsp5A) lines from vegetative to generative phase. In red are the compounds with increased concentrations upon the transition from the cold-treated vegetative stage to the double ridge formation stage (Co-Dr) and from the double ridge stage to the spikelet formation stage (Dr-Sp). In green are the compounds with decreased concentrations, while in black are the compounds with unchanged concentrations comparing the CS(Ch5A) to CS and the CS(Tsp5A) to CS. The grey letters depict the compounds that were not detected in the pathway. The concentration changes in shoots are circled, while those in the crowns are not circled. The concentration differences resulting from an increase in the CS but not in the CS(Ch5A) shoots are indicated by black letters and red circles. The concentration differences resulting from an increase in the shoots but a decrease in the crowns of CS(Tsp5A) are indicated by green letters and red circles. The differences resulting from a decrease in the CS crowns are depicted by green underlines. The schemes are based on the data that were presented in Figure 4. [file 12870_2014_363_MOESM8_ESM.pptx]
